# Identifying priority areas for conservation to promote connectivity and mitigate the impacts of anthropogenic disturbance

**DOI:** 10.1111/cobi.70083

**Published:** 2025-05-31

**Authors:** Edmond Sacre, Ulf Bergström, Charlotte Berkström

**Affiliations:** ^1^ Department of Aquatic Resources Swedish University of Agricultural Sciences Öregrund Sweden; ^2^ Department of Aquatic Resources Swedish University of Agricultural Sciences Uppsala Sweden

**Keywords:** anthropogenic disturbance, ecological coherence, ecological connectivity, marine protected areas, pressures, protected area networks, spatial prioritization, systematic conservation planning, áreas marinas protegidas, coherencia ecológica, conectividad ecológica, planeación sistemática de la conservación, perturbación antropogénica, presiones, priorización espacial, redes de áreas protegidas, 空间优先保护, 系统性保护规划, 生态连通性, 人为干扰, 压力, 海洋保护区, 保护区网络, 生态连贯性

## Abstract

As nations seek to expand protected area (PA) networks to cover 30% of land and seas by 2030 (30×30), there is an urgent need for systematic conservation planning and spatial prioritization that considers the broad range of ecological and socioeconomic factors influencing the persistence of biodiversity. A remaining challenge in spatial prioritization is identifying areas that not only contribute to ecological connectivity but also are vulnerable to isolation and connectivity decline caused by anthropogenic disturbance. We devised an approach to assess PA networks and prioritize areas for conservation action and applied it to the Swedish coastal Baltic Sea area as an example. We developed connectivity models for 16 key fish species to identify habitats that provide the greatest contributions to maintaining network connectivity. We then incorporated spatial data on anthropogenic disturbance into the connectivity models to identify habitats for which human activities may hinder dispersal and recruitment, making them vulnerable to local population declines. We assessed the adequacy of the marine protected area (MPA) network in protecting these biodiversity features. Using spatial prioritization with explicit objectives to protect these biodiversity features, we then identified important areas for future protection. Although the Swedish MPA network provided a reasonable level of protection for these key habitats, their protection in stricter MPA categories (International Union for Conservation of Nature categories Ia, Ib, and II) was poor. Expanding the MPA network from its current coverage (10.5% of the study area) to 11%, the mean protection level across features increased from 25% to 48%. Expanding to 15% coverage increased mean protection across features to over 90%. Our approach to conservation planning incorporated not only biodiversity data (e.g., habitats and connectivity) but also the pressures these elements of biodiversity are susceptible to from human activities.

## Abstract (es)

Identificación de las áreas prioritarias para la conservación para promover la conectividad y mitigar el impacto de la perturbación antropogénica

**Resumen:**

A medida que los países intentan ampliar las redes de áreas protegidas para cubrir el 30% de la tierra y los mares en 2030 (30 x 30), surge la necesidad urgente de una planificación sistemática de la conservación y una priorización espacial que tenga en cuenta la amplia gama de factores ecológicos y socioeconómicos que influyen en la persistencia de la biodiversidad. Un reto pendiente en la priorización espacial es la identificación de áreas que no sólo contribuyen a la conectividad ecológica, sino que también son vulnerables al aislamiento y al declive de la conectividad causados por perturbaciones antropogénicas. Hemos ideado un enfoque para evaluar las redes de áreas protegidas y priorizar las zonas que requieren medidas de conservación, y lo hemos aplicado a la zona costera sueca del Mar Báltico a modo de ejemplo. Desarrollamos modelos de conectividad para 16 especies de peces clave con el fin de identificar los hábitats que más contribuyen a mantener la conectividad de la red. Después, incorporamos datos espaciales sobre perturbaciones antropogénicas a los modelos de conectividad para identificar los hábitats en los que las actividades humanas pueden dificultar la dispersión y el reclutamiento, lo que los hace vulnerables al declive de las poblaciones locales. Evaluamos la idoneidad de la red de áreas marinas protegidas (AMP) para proteger estas características de la biodiversidad. Mediante la priorización espacial con objetivos explícitos de protección de estas características de biodiversidad, identificamos áreas importantes para su futura protección. Aunque la red sueca de AMP ofrecía un nivel razonable de protección de estos hábitats clave, su protección en categorías de AMP más estrictas (categorías Ia, Ib y II de la Unión Internacional para la Conservación de la Naturaleza) era deficiente. Al ampliar la red de AMP de su cobertura actual (10.5% de la zona de estudio) al 11%, el nivel medio de protección de todas las características aumentó del 25% al 48%. La ampliación a una cobertura del 15% aumentó la protección media de las características a más del 90%. Nuestro enfoque de la planificación de la conservación incorporó no sólo datos sobre biodiversidad (por ejemplo, hábitats y conectividad), sino también las presiones a las que estos elementos de la biodiversidad son susceptibles de ser sometidos por las actividades humanas.

## 确定优先保护区域以提高连通性并减缓人为干扰的影响

【摘要】

随着各国都在努力扩大保护区网络, 从而在2030年前保护30%的陆地和海洋(30 x 30目标), 目前迫切需要进行系统性保护规划和空间优先保护规划, 以考虑影响生物多样性续存的各种生态和社会经济因素。在空间优先保护规划方面的一个现存挑战在于, 确定哪些区域不仅有利于提高生态连通性, 而且还容易受到人为干扰造成的隔离和连通性下降的影响。我们设计了一种评估保护区网络并确定保护行动优先区域的方法, 并将其应用于瑞典波罗的海沿岸地区开展了案例研究。我们为16种关键鱼类开发了连通性模型, 以确定对维持网络连通性贡献最大的栖息地。接下来, 我们在连通性模型中纳入了人为干扰的空间数据, 以确定人类活动可能会阻碍鱼类扩散和种群繁殖, 并导致其易受局部种群数量下降影响的栖息地。我们还评估了海洋保护区(marine protected area, MPA)网络是否能充分保护这些生物多样性特征。通过对明确保护这些生物多样性特征的目标进行空间优先规划, 我们确定了未来需要保护的重要区域。尽管瑞典MPA网络已为这些关键栖息地提供了合理水平的保护, 但在更严格的MPA类别(国际自然保护联盟Ia、Ib和II)中, 保护情况却不容乐观。当将MPA网络的覆盖度从目前(本研究区域的10.5%)扩大到11%时, 各个生物多样性特征的平均保护水平将从25%提高到48%。当将覆盖度扩大到15%时, 各个特征的平均保护水平能提高至90%以上。我们的保护规划方法不仅纳入了生物多样性数据(如栖息地和连通性), 还纳入了这些生物多样性要素是否易受人类活动影响的压力情况。**【翻译:胡怡思;审校:聂永刚】**

## INTRODUCTION

As Earth's ecosystems experience mounting anthropogenic pressures, conservation scientists and practitioners are faced with the task of developing strategies to prevent the loss of biodiversity and restore ecosystem functions. One of the most widely used management measures for limiting the overexploitation of natural resources and promoting population recovery is protected areas (PAs) (Geldmann et al., 2019; Maxwell et al., 2020; Visconti et al., 2019). However, PAs are often implemented opportunistically based on circumstantial factors, such as community willingness and desire to conserve and manage specific areas, and the absence of conflicting activities (Margules & Pressey, 2000). This nonsystematic, ad hoc approach fails to consider broader ecological patterns and processes, such as landscape‐scale habitat distribution and connectivity, which can lead to PA networks with a questionable capacity to preserve populations and ecosystems (Pressey et al., 2015, 2021).

To remedy this problem, conservation planners have developed systematic approaches to the design of PA networks in the field of systematic conservation planning (SCP). One step in the process of SCP is spatial prioritization, which is the identification of locations where management actions would be an efficient use of the limited resources available for conservation to maximize benefits to biodiversity and society (Kukkala & Moilanen, 2013; Margules & Pressey, 2000; Tallis et al., 2021). Various approaches to spatial prioritization have been developed within SCP, such as ensuring the representation of species and habitats (Ballantine, 2014; Fernandes et al., 2005; Saarman et al., 2013) and maximizing connectivity (Jacobi & Jonsson, 2011; Krueck et al., 2017; Magris et al., 2018; White et al., 2014). However, simultaneous advancements in SCP have drawn attention to the importance of quantifying not only the ecological features and processes in PA networks but also the anthropogenic pressures that threaten these ecosystems (Albert et al., 2017; Crook et al., 2015; Kang et al., 2024). Failure to consider pressures in conservation planning runs the risk that PA networks will exhibit residual patterns, which is a bias in the location of PAs toward areas of low economic value where conflict with human activities is minimal (Devillers et al., 2015, 2020; Hoekstra et al., 2005; Joppa & Pfaff, 2009; Pressey et al., 2015; Vieira et al., 2019).

Of particular importance in the context of SCP is the interaction between ecological connectivity and anthropogenic pressures. Ecological connectivity is typically defined as the movement of energy, organisms, or genes between locations (Beger et al., 2022). In recent years, it has become apparent that, given the influential nature of connectivity on populations and, consequently, the ecosystems they inhabit, consideration of connectivity is essential to achieve positive outcomes from conservation actions (Fontoura et al., 2022; Magris et al., 2018). Anthropogenic pressures may affect connectivity through disturbance or destruction of source and sink areas, by direct interference with dispersing propagules or migrating adults (e.g., dredging and fishing), or by affecting dispersal movements (e.g., physical structures or changes in hydrodynamics and passive dispersal of aquatic organisms [Crook et al., 2015]). In such cases, if connectivity is compromised, then recolonization of disturbed areas can be limited and lead to local extinctions and loss of ecosystem functions and, as a consequence, cause broader patterns of low genetic diversity and population decline (Staddon et al., 2010).

We devised an approach to spatial connectivity modeling and conservation prioritization that quantifies the contribution of species habitats to overall network connectivity and the vulnerability of habitats to isolation and decline in connectivity caused by anthropogenic disturbance. Our overarching goal was to identify important areas for the conservation of demographic connectivity processes that underpin short‐term (i.e., years or decades) recruitment rates and population persistence (Kendrick et al., 2017). To do so, we produced models of ecological connectivity and vulnerability for 16 ecologically and economically important coastal fish species in the Swedish Baltic Sea. We then assessed the degree to which these aspects of connectivity are protected in the existing marine protected area (MPA) network in the region. Finally, we performed a spatial prioritization analysis under a scenario in which the existing MPA network is expanded to improve protection of these features. In doing so, we sought to identify priority areas for the protection of habitats, important connectivity sources, and habitats vulnerable to connectivity decline and isolation.

## METHODS

### Study area

We focused on the coastal Swedish Baltic Sea, an area of 49,469 km^2^, covering a coastline approximately 1500‐km long. The study area was defined as all sea areas within 15 km of the Swedish baseline, a line joining the outermost Swedish islands as defined by the Swedish Maritime Administration. The Baltic Sea is a large brackish water basin that exhibits strong environmental gradients. A strong north–south salinity gradient exists, with waters in the north having low salinities and waters in the south having higher salinities, which is driven by saltwater inflows in the south through the Danish straits (Lehmann et al., 2022). Similarly, a strong latitudinal gradient in temperature and seasonality is also evident, with cooler water temperatures and winter ice cover in the north (Dutheil et al., 2023). Strong coastal–offshore gradients also exist, which are driven largely by the presence of extensive archipelagos, including the Swedish–Finnish archipelago, which contains the largest number of islands of any archipelago in the world. The archipelagos are extremely heterogeneous in terms of wave exposure, water temperature, turbidity, and, to some extent, salinity (Sundblad et al., 2009). The strong gradient in salinity allows both freshwater‐adapted and saltwater‐adapted species to inhabit the Baltic Sea, which, in turn, has led to the formation of unique species assemblages, which vary along these gradients (Bonsdorff, 2006; Koehler et al., 2022).

As in much of the world, coastal areas in the Baltic Sea are popular locations for human settlement and exploitation. As such, near‐shore marine ecosystems are affected by a variety of anthropogenic pressures, such as coastline development, boating, fishing, toxicants and pollutants, invasive species, and eutrophication (Brown et al., 2018; Hansen et al., 2019; Sundblad & Bergström, 2014). Eelgrass (*Zostera marina*) meadows, for example, can be directly removed by dredging, or can be disturbed by shading (reduced light availability) caused by nearby structures, such as jetties and marinas (Eriander et al., 2017). Similarly, disturbance by ships, ferries, and recreational boats (e.g., anchoring, waves, and currents) can directly affect important vegetation crucial to fish spawning and recruitment (Hansen et al., 2019). In Sweden, these pressures pose a particular problem given the growing population and the situation of many of its large cities and towns along the coast.

### Connectivity models and matrices

**FIGURE 1:**
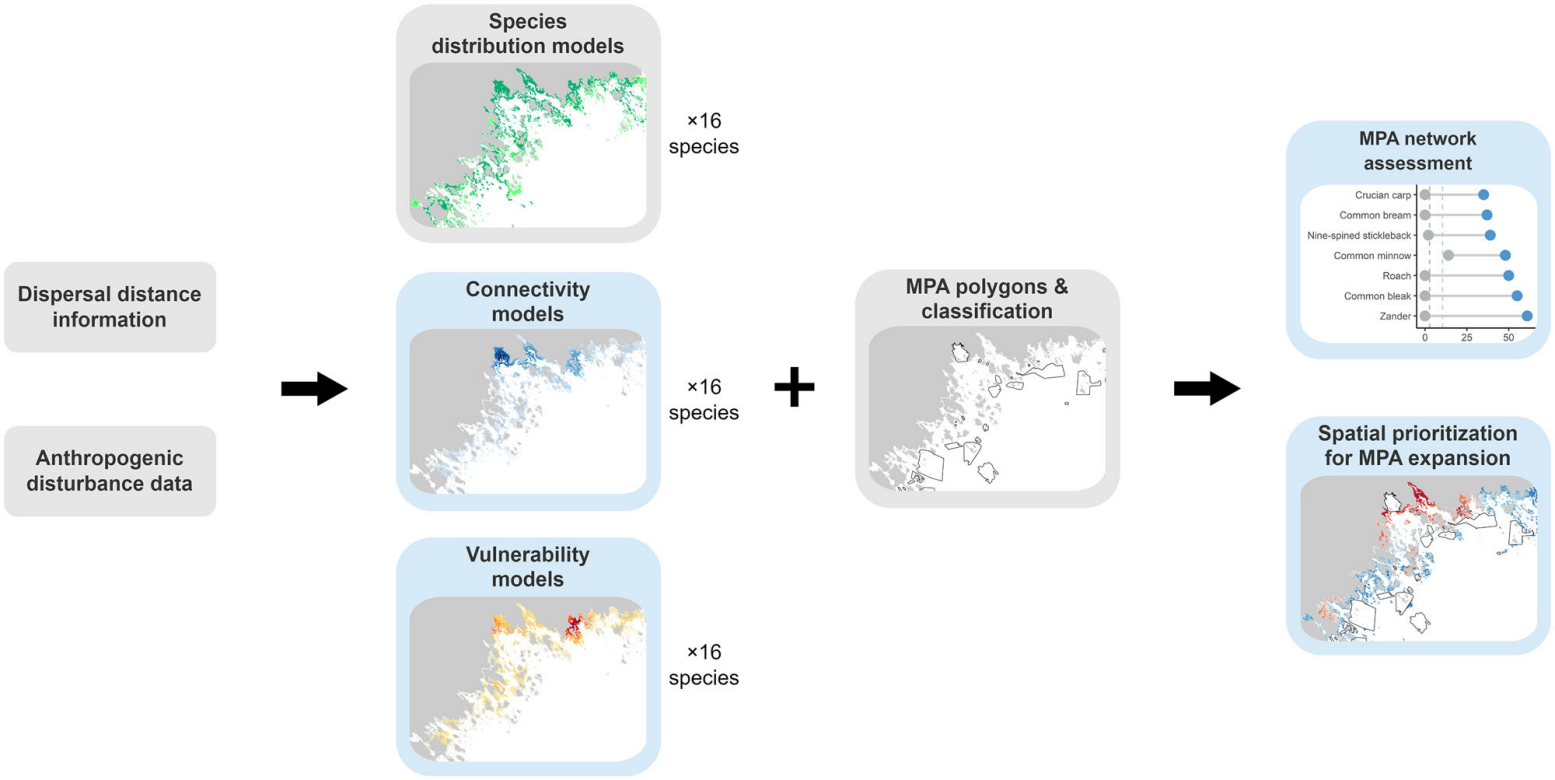
Analyses and modeling workflow (blue boxes, products or analyses produced in this study; gray boxes, information or data obtained from other studies or databases; MPA, marine protected area).

As a basis for our spatial estimation of different aspects of connectivity, we developed connectivity models and matrices that describe the pairwise connectivity between species habitats in the region. A flow chart describing all models and analyses is provided in Figure 1. We developed connectivity models for 16 fish species present in the coastal Swedish Baltic Sea (Appendix S1). The connectivity models (and all subsequent analyses) were produced at a resolution of 250 m, totaling 791,511 grid cells across the study region. The vast majority of coastal fish species in the Baltic Sea have short larval phases and instead disperse primarily through active movements during the juvenile or adult phases, meaning dispersal movements for these species are not greatly influenced by hydrodynamics (Berkström et al., 2022). As such, active dispersal during the juvenile or adult phases is the primary driver of spatial connectivity patterns of fish in the study region. The species selected for inclusion in the analyses were chosen because they are common in coastal fish communities and their primary mode of dispersal is active movements over relatively short distances (1–20 km). Species that perform long dispersal movements, such as herring and flounder, were not included because their high mobility generates highly connected and uniform networks across areas larger than our study area. Planning for these species, therefore, is more relevant at larger spatial scales, such as the entire Baltic Sea.

The connectivity models were developed using a degree centrality approach based on graph theory. Degree centrality measures the number of connections or edges each node in a network has to other nodes in the network. In ecological studies, a node is typically represented by a discrete habitat patch. However, the drawback to this approach is that it does not capture the continuous nature of habitats and habitat quality (Moilanen, 2011). In certain cases, such as with coral reefs, this is appropriate because it is generally clear which areas are and are not coral reef habitat. However, for many species, particularly fish in the Baltic Sea, habitats occur on a gradient from highly suitable to highly unsuitable. In our models, therefore, we included all raster cells containing habitat as nodes in the analysis. As such, the strength of the connection between habitat cells for each species was measured as a function of habitat suitability in a given cell, the least‐cost path distance to other habitat cells, and a negative exponential dispersal kernel. Specifically, for a given species, the connectivity export, *k*, from habitat cell *i* to habitat cell *j* is defined by

(1) $$k_{i,j}=\left\{\begin{array}{c} \exp\left(-1\frac{d_{i,j}}{\alpha d_{\max}}\right)s_{i},\;\;\;d_{i,j}\leq d_{\max},\\ 0,\;\;\;\;\;\;\;\;\;\;\;\;\;\;\;\;\;\;\;\;\;\;\;\;\;d_{i,j}>d_{\max}, \end{array}\right.$$

where α is a constant that dictates the steepness of the exponential decay of the dispersal kernel, *d_i_*_,_ *_j_* is the distance between habitat cells *i* and *j* along the least‐cost path, *d*_max_ is the maximum dispersal distance for a given species, and *s_i_* is the habitat quality of habitat cell *i*. If the distance along the least‐cost path between cells is greater than the defined maximum dispersal distance, then *k_i_*_,_ *_j_* = 0. We assumed a value of α = 0.3 for all analyses presented in this study, which provides a moderately steep decline in connectivity with distance (Appendix S1). We chose a value of 0.3 because it aligns with behaviors observed through mark–recapture studies of coastal fish species in the Baltic Sea, which most commonly perform shorter dispersal movements but occasionally travel longer distances (Olin et al., 2024; Saulamo & Neuman, 2002). In all models, land was treated as an obstacle to migration and could not be travelled through in calculations of the least‐cost path. Least‐cost paths were calculated using the costDistance function in the gdistance package (van Etten, 2017) in R (R Core Team, 2024) with a uniform cost layer with a value of 1 in each cell. As such, the distance between habitat cells represents the distance along the least‐cost path (the shortest path) through cells containing water.

Connectivity values were calculated for each habitat cell and each species to produce a connectivity matrix, one for each species, describing the pairwise connectivity exports and imports between habitat cells. The matrices, therefore, are square, with the number of columns and rows being equal to the number of habitat cells. In these connectivity matrices, rows (*i*) represent the connectivity donors (producing connectivity exports) to all other habitat cells, and columns (*j*) represent the connectivity recipients (receiving connectivity imports) from all other habitat cells. When habitat quality is the same in all habitat cells, the connectivity matrix is symmetrical because connectivity exports and imports are of equal strength. If habitat quality varies, the matrix is nonsymmetrical because habitat quality only affects connectivity exports.

Maximum dispersal distances for each species were determined based on information from the scientific literature. A full list of sources and justification for the chosen maximum dispersal distances is provided in the Supporting Information (Appendix S1). All models were implemented in R 4.4.0. The code for the connectivity models is available as an R package at https://github.com/EdSacre/wandeR (see the connect function). To illustrate the functionality of the connectivity models, the relationship between connectivity values and the number of connections is provided in Appendix S1.

As species habitat maps in our connectivity models, we used ensemble species distribution models produced by Erlandsson et al. (2021). These models predict nursery habitats, which constitute a bottleneck for coastal fish populations in the Baltic Sea and are particularly sensitive to human disturbance (Sundblad & Bergström, 2014). The models were based on field observations of the occurrence of juvenile fish in combination with information on a set of environmental predictor variables at sampling locations. Erlandsson et al. (2021) divided the predicted habitats into 3 categories of habitat quality: absent, good quality, and very good quality. When using these habitat maps as inputs in the connectivity models, the respective habitat qualities were assigned values of 0.0, 0.5, and 1.0.

### Habitat importance for network connectivity

**FIGURE 2:**
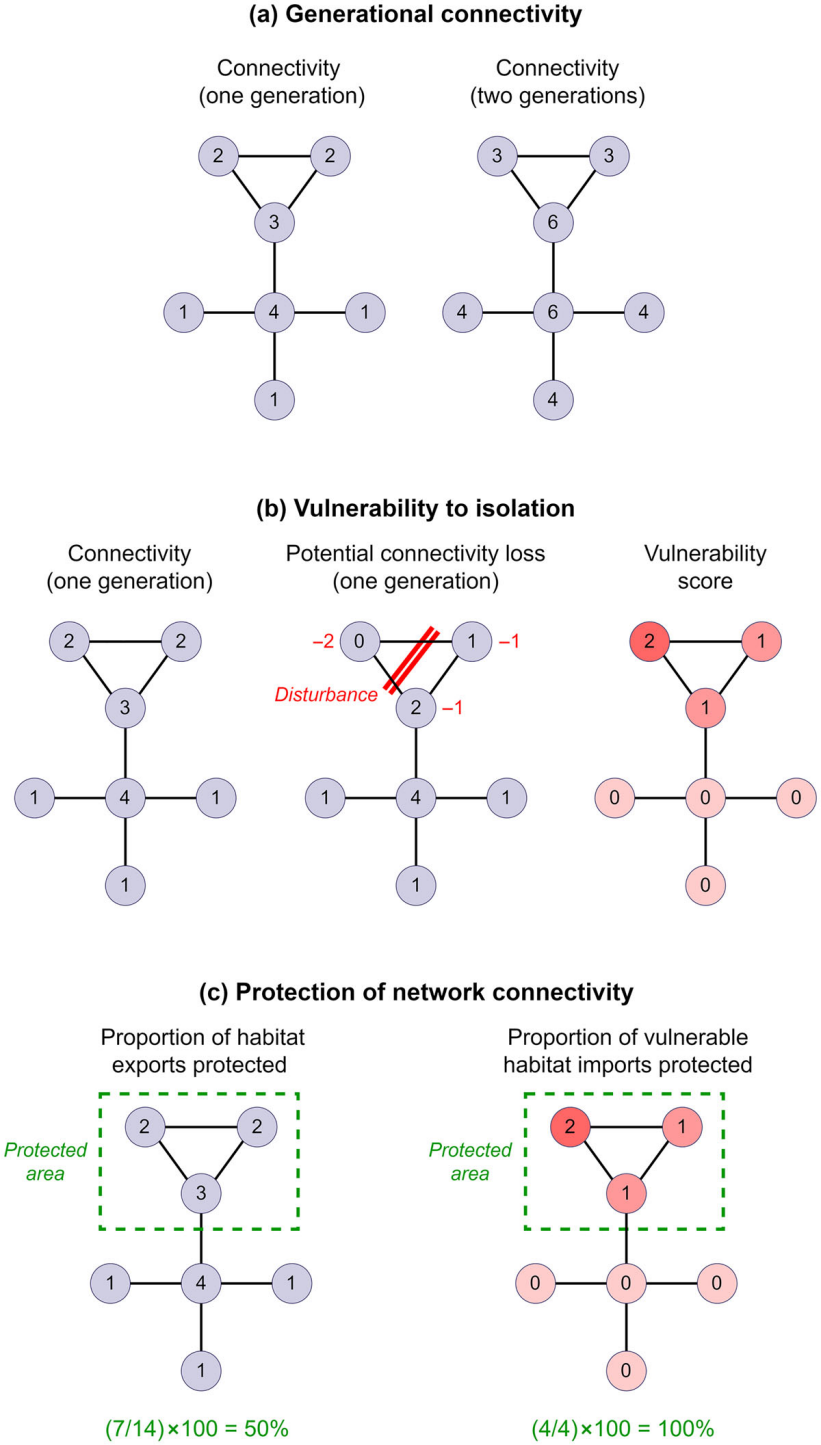
Connectivity models and methods for assessing a hypothetical habitat network with 7 nodes (i.e., habitat cells) (numbers in nodes, number of connections the node has with other nodes in the network; lines, connected nodes within the maximum dispersal distance of one another): (a) connectivity over one and 2 generations or dispersal events in, for simplicity, a network in which all nodes are of equal habitat quality and so connectivity exports and imports are the same and produce a symmetrical connectivity matrix, although when habitat quality varies spatially, the connectivity matrix is likely nonsymmetrical and thus exports and imports can be unequal; (b) how habitat vulnerability to isolation is measured in the models (vulnerability score, absolute loss of connectivity imports in each node when disturbance is incorporated into the model); and (c) how the marine protected area network is assessed in terms of its capacity to protect connectivity (left side, proportion of connectivity exports in the network coming from protected habitats; right, proportion of vulnerable connectivity imports predicted to occur in protected habitats). For simplicity, connectivity values are the number of connections to other nodes, but in our case study, connectivity values were calculated according to a dispersal kernel.

To estimate the contribution of each species habitat cell to network connectivity (henceforth referred to as habitat connectivity), we measured the quantity of connectivity exports from each habitat cell to other habitat cells in the region (Figure 2). In our models, the quantity of connectivity exports represented the cumulative probability of individuals from a given habitat reaching other habitats. However, when the input habitat maps represented species abundances, exports could be interpreted as the predicted number of individuals exported to other habitat cells. Habitats with a large number of exports represented important source habitats that contributed substantially to recruitment and survival of the metapopulation (Almany et al., 2009; Bode et al., 2006).

We measured habitat connectivity over 3 generations (or dispersal events), which provides a useful measure of short‐term demographic connectivity processes (e.g., years or decades). This approach captured potential stepping‐stone habitats that may produce minimal connectivity exports but may connect important source habitats. The generational connectivity matrix was produced by multiplying each connectivity matrix by itself 3 times (with matrix multiplication). The total habitat connectivity was then calculated as the row sums in the generational connectivity matrix. In the supporting web application (https://eclive.shinyapps.io/sweconnect/), model outputs over one and 2 generations are provided.

### Models of habitat vulnerability to connectivity decline and isolation

To identify habitats vulnerable to decline in connectivity and isolation (henceforth referred to as vulnerable connectivity), we quantified the extent to which the connectivity imports to each species habitat are potentially hindered by anthropogenic disturbance. The reason we used connectivity exports to measure habitat connectivity importance and connectivity imports to measure vulnerability was that habitats are likely to be at risk of isolation and local population decline when the import of individuals is low. This could potentially limit external recruitment and repopulation of the habitat if it is disturbed (e.g., fishing, destruction, or extreme weather events). In contrast, connectivity exports are more useful for determining the importance of habitats for the maintenance of the network and population through recruitment in connected habitats.

We used Törnqvist et al.’s (2020) map of anthropogenic disturbance that could affect connectivity, which was produced for Swedish coastal areas based on human structures and activities, including dumping, dredging, piers, marinas, ports, and jetties. In Törnqvist et al.’s (2020) map, the likelihood of disturbance impeding connectivity was classified into 5 categories based on the intensity and density of activities. A value of 5 represented the highest level of obstruction of the movement of individuals. This obstruction potential is defined by Törnqvist et al. (2020) as the “disruption of species’ ability or propensity to move across areas in a natural way, through the presence of obstacles, noise, and altered water flows” (translated from Swedish). Using these data, we generated a second set of connectivity models for all species, in which the anthropogenic disturbance zones were included as an additional resistance in the calculation of the least‐cost paths. Areas were assigned a probability of successful passage based on their zone. Zones 1–5 were assigned a probability of 50%, 40%, 30%, 20%, and 10%, respectively. These values should not be interpreted as real‐world probabilities, but rather as assumed values that represent relative differences between levels of disturbance.

We incorporated the probabilities into the models by setting conductance values (the inverse of cost) in the calculation of the least‐cost paths to 0.5, 0.4, 0.3, 0.2, and 0.1, respectively. As such, cells containing physical disturbance have a higher cost to travel through (or lower conductance), which reduces connectivity (*k*) between habitat cells. Following the same method as above, a connectivity matrix was then produced describing the pairwise connectivity between habitat cells for each species. Thus, 2 connectivity matrices were produced for each species, one without disturbance included in the model (Matrix A) and one with disturbance included in the model (Matrix B). Connectivity values in each matrix were then transformed to represent connectivity over 3 generations (or dispersal events) by multiplying each connectivity matrix by itself 3 times. To calculate the connectivity imports to habitats in the base connectivity model and the connectivity model including anthropogenic disturbance, we calculated the column sums in each generational matrix for each species. Finally, for each species, we subtracted the column sums in the matrix including disturbance (Matrix B) from the column sums in the base matrix (Matrix A) and took the absolute value of this difference to yield an estimate of the change in connectivity imports caused by disturbance. Because there are little observational data on how anthropogenic disturbance affects dispersing individuals, these habitat vulnerability scores should be interpreted as a relative spatial index. We also provide vulnerability model outputs over one and 2 generations in the supporting web application (https://eclive.shinyapps.io/sweconnect/).

### Assessment of the MPA network

We assessed the effectiveness of the Swedish MPA network based on the following 3 criteria: proportion of habitat connectivity exports protected, proportion of vulnerable connectivity protected, and proportion of habitat protected (i.e., based on the species habitat maps used as inputs for the connectivity models). We included the proportional protection of the base habitat maps because habitats (regardless of connectivity) serve an important ecological role in the local community, such as the provision of prey for other species or predators that regulate food web dynamics (Williams et al., 2020). The proportions for each of the criteria were measured for each species. Figure 2 illustrates how these proportions were measured in relation to habitat connectivity and vulnerability.

Spatial polygons of MPA boundaries were obtained from the Swedish Environmental Protection Agency protected area database (Swedish Environmental Protection Agency, 2022). We measured the proportional protection of features in strict MPAs and all MPAs (strict and not strict). Strict MPAs were defined according to the European Commission's guidelines for PA designation, which suggest that MPAs designated as International Union for Conservation of Nature (IUCN) categories Ia, Ib, and II can be considered strictly protected (European Commission, 2022). Thus, all MPAs designated in those categories were defined as strict in the present analysis. Any MPAs that did not have an IUCN designation (approximately 33% of the area protected in the study region) were assumed not to be strictly protected. If an area was covered by multiple MPAs with different designations, we assumed the strictest protection level, as the presence of other less strict regulations from overlapping MPAs does not override strict regulations. The code for this and all aforementioned analyses is openly available at https://github.com/EdSacre/swe‐connect.

We compared protection levels for each species and criterion with expected protection levels from random allocation of MPAs. The total area covered by the full MPA network in the study area was 5200 km^2^, which constitutes approximately 10.5% of the study area. The total area covered by strict MPAs was 1373 km^2^, which constitutes approximately 2.8% of the study area. As such, random allocation of MPAs would be expected, on average, to cover 10.5% of each biodiversity feature (habitat connectivity, habitat vulnerability, and habitat), whereas random allocation of strict MPAs would be expected to cover 2.8% of each feature. Protection levels exceeding random expectation levels indicated a positive bias toward prioritizing features, whereas protection levels falling short of random expectation indicated a negative bias toward avoiding the protection of features.

### Spatial prioritization for expansion of the MPA network

We identified spatial priorities for the expansion of the MPA network in Sweden with the same 3 features used in the MPA network assessment: habitat connectivity exports, habitat vulnerable connectivity, and habitat. The prioritization included each of the 3 aforementioned feature types for each of the 16 species, totaling 48 features. For the spatial prioritization, we assumed that existing MPAs were locked in, meaning they must be included in the prioritization. This provides a more useful output for management because it would likely be challenging to move or remove existing MPAs. Instead, we showed areas where MPAs could be added to the network to maximize protection of the aforementioned features.

To implement the prioritization, we used the prioritizr R package (Hanson et al., 2017) with the Gurobi optimizer (Gurobi Optimization, LLC, 2023). The prioritizr package (when used with Gurobi) is a conservation planning tool that can identify mathematically optimal areas for protection according to specified conservation features (e.g., species connectivity or distribution maps) and targets (e.g., protect 30% of each feature). Our prioritization was implemented using the minimum shortfall objective, for which the objective was to protect 100% of every feature within a specified budget. We used a homogeneous cost layer, where the cost of protection of each planning unit (grid cell) was set to one. The budget and costs, therefore, were considered proportional to the area of the solution, and solutions covered large areas requiring large budgets. The planning problem contained 791,511 planning units.

To identify priority areas, we iteratively increased the budget in increments of 1%, starting with 11% of the study area (a budget of 87,066), which is slightly larger than the coverage of the existing MPA network. This iterative process was repeated until all objectives were met (i.e., 100% of all features for all species are protected). Planning units were then assigned a priority ranking based on which increment they were selected in, with planning units selected in the first increment (11% budget) receiving the highest ranking, planning units selected in the second increment (12% budget) receiving the second highest ranking, and so forth. This approach to identifying priority or importance is useful when the planning problem contains a large number of planning units and features, as is the case in our analyses. However, other methods, such as irreplaceability scores, are likely superior, but calculations of irreplaceability are extremely computationally intensive and were not feasible to measure for our planning problem.

## RESULTS

### Assessment of the Swedish MPA network

**FIGURE 3:**
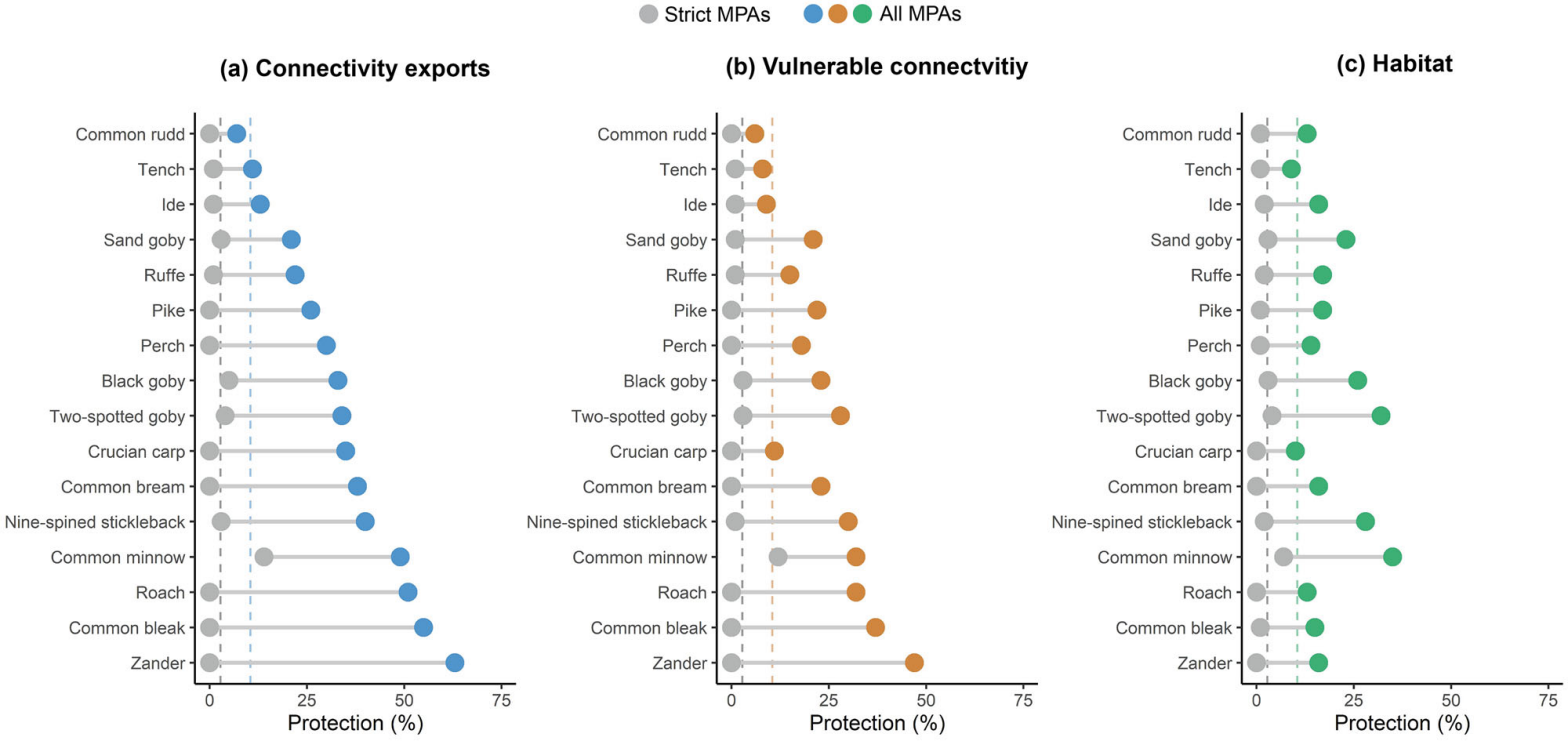
Levels of protection of (a) connectivity exports, (b) vulnerable connectivity, and (c) habitat in the Swedish marine protected area (MPA) network (blue, green, and orange circles, protection levels in the full MPA network; gray circles, protection levels in strict MPAs [IUCN categories Ia, Ib, and II]; blue, green, and orange dashed lines, 10.5% protection [i.e., coverage of the current MPA network and the expected protection level from random allocation of MPAs]; gray dashed lines, 2.8% protection [coverage of strict MPAs and the expected protection level from random allocation of strict MPAs]).

Habitat connectivity was reasonably well protected in the full MPA network (Figure 3a) (mean protection level of 33.0% across species). This exceeds the protection levels expected from random allocation of protection, which would be 10.5% (the present‐day coverage of the MPA network). Of the 16 species in our analyses, 15 had protection levels exceeding random expectation in the full MPA network. Habitat connectivity was not well protected by strict MPAs; the mean protection level was 1.8% across species. The expectation from random allocation was 2.8%. In the strict MPA network, only 4 of the 16 species had protection levels exceeding random expectation.

Vulnerable connectivity was also reasonably well protected in the full MPA network (Figure 3b) (mean protection level 22.6% across species). Thirteen of the 16 species had protection levels exceeding random expectation. Protection of vulnerable connectivity was poor in strict MPAs (mean protection level 1.5%). Only 2 of the 16 species had protection levels for vulnerable connectivity exceeding random expectation.

Habitats were less well protected than the other features (mean protection level of 18.7% across species) (Figure 3c). Fourteen of the 16 species exceeded expectation from random allocation. As with the other features, habitats were not well protected with strict MPAs (mean protection level of 1.8%). Only 4 of the 16 species had habitat protection levels exceeding random expectation in strict MPAs.

### Priority areas for expansion of the MPA network

In the incremental priority expansion analysis, all objectives were achieved (100% protection of all features) when the budget reached 20% of the study area. In other words, total protection of all features was achieved by doubling the size of the current MPA network when areas selected in the prioritization were protected. There were diminishing returns on the mean protection level of features as the budget increased. The point of inflection occurred around a budget of approximately 13–14% of the study area (Figure 3). The mean protection of all features exceeded 90% with a budget of 15% of the study area. The initial expansion of the MPA network from 10.5% (the current extent of the MPA network) of the study area to 11% of the study area increased the mean protection across all features from 24.7% to 48.1% (Figure 3).

**FIGURE 4:**
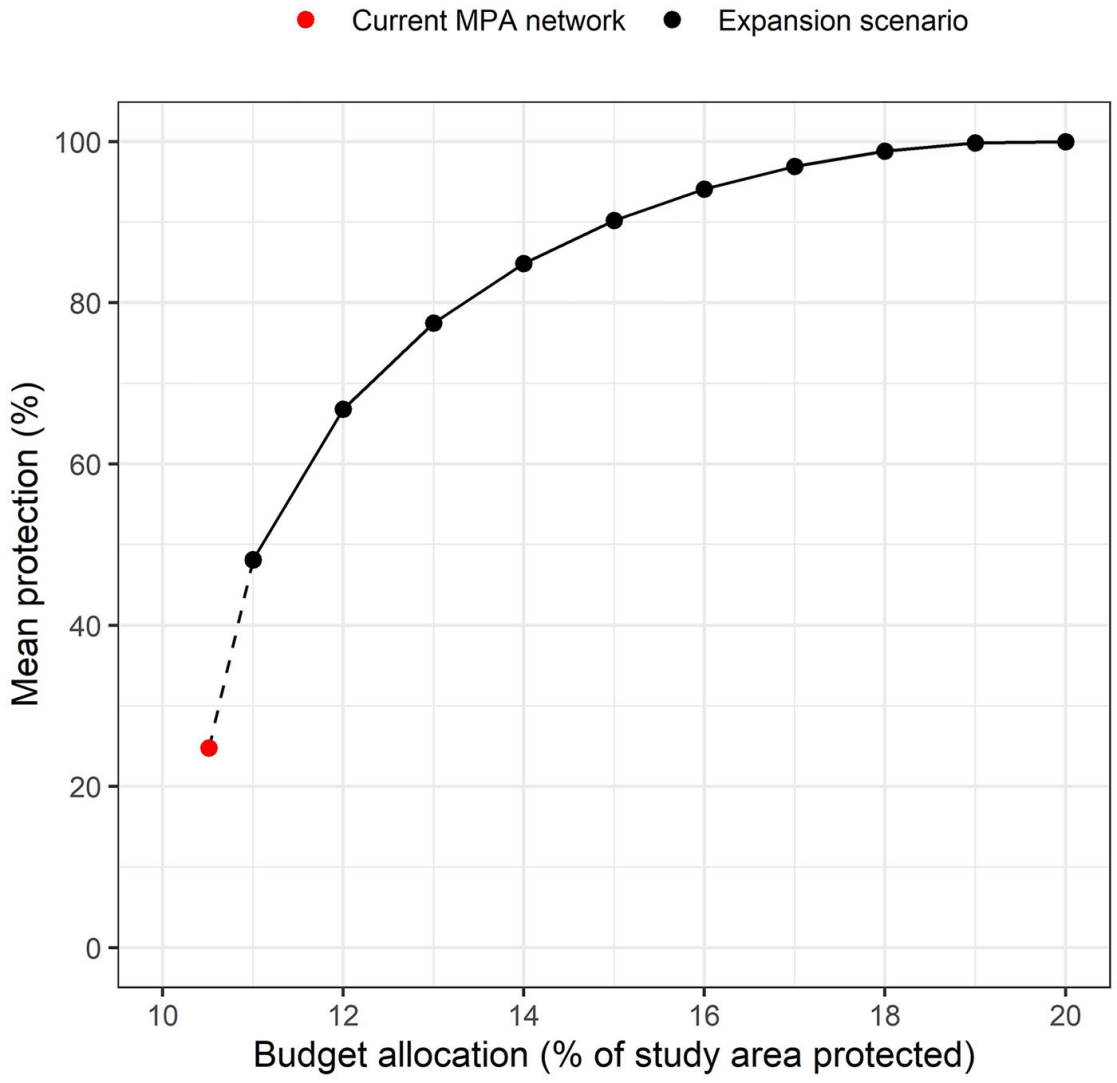
Mean percent protection across all biodiversity features (48 features) achieved in the incremental Swedish marine protected area (MPA) expansion scenario relative to budget allocations as the percentage of the total area protected (red point, extent and mean percent protection across all features in the current MPA network in the study region). Because all planning units were assigned a cost of 1, this is equivalent to the percentage of planning units protected in the solution.

**FIGURE 5:**
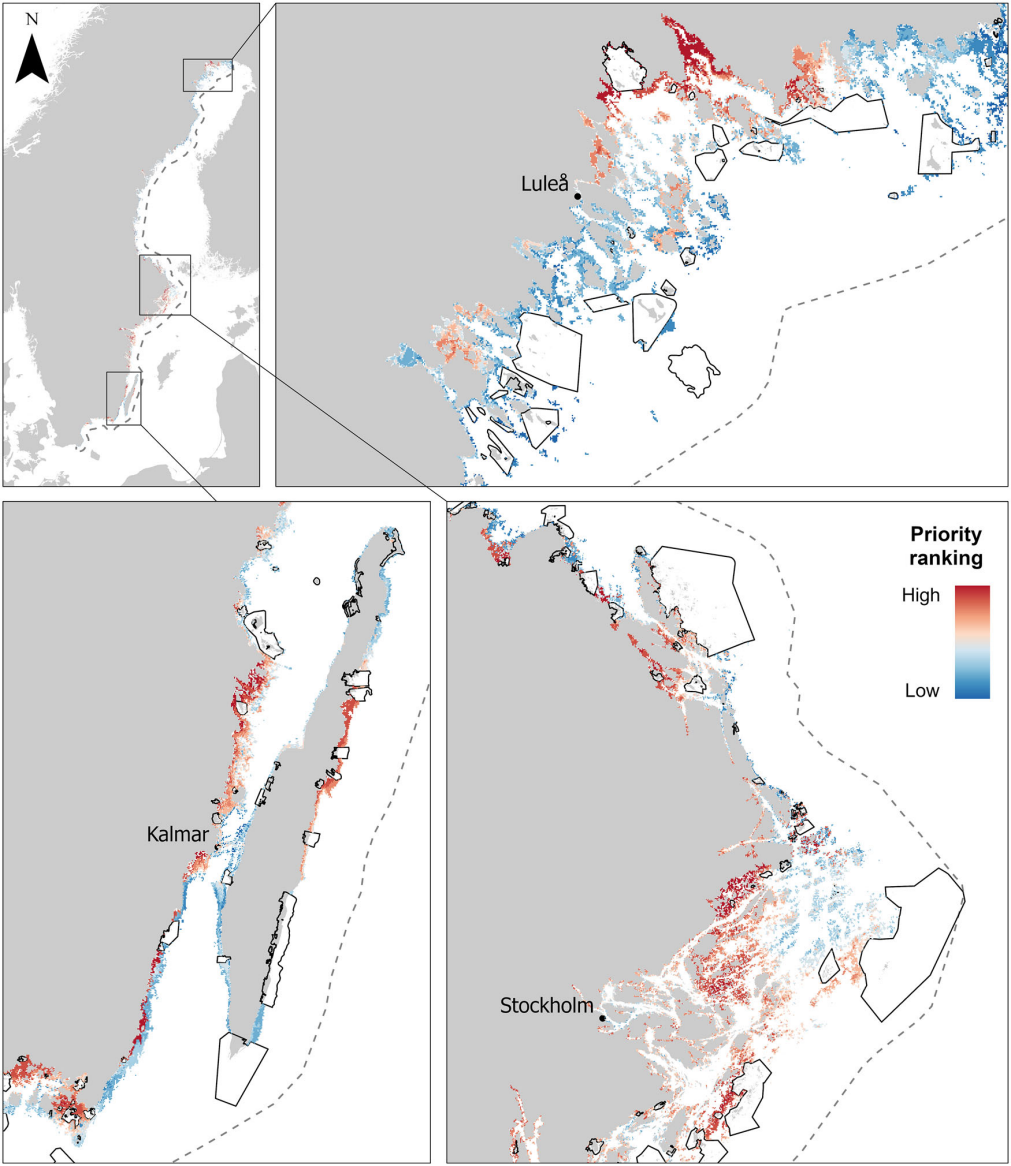
Priority areas for expansion of the Swedish marine protected area (MPA) network to protect habitats, connectivity, and vulnerable connectivity for 16 fish species (dark red, areas of high priority for protection; dark blue, habitats that should be protected after high‐priority areas are secured; black polygons, current Swedish marine protected areas; dashed line [top left], extent of the study region). Areas in existing MPAs are part of the solution and, therefore, do not have a priority score. Only MPAs that intersect the study area are shown in the figure.

In general, priority areas occurred in shallow, sheltered areas with low wave exposure, particularly in the central and southern Baltic (Figure 4). This is likely because the species in our analyses prefer such habitats and because such areas are also subject to high levels of anthropogenic disturbance (Brown et al., 2018). Deeper and more exposed areas were rarely selected as a priority. Of all species, ruffe (*Gymnocephalus cernuus*), sand goby (*Pomatoschistus minutus*), and 2‐spotted goby (*Pomatoschistus flavescens*) were the last to reach full protection in the prioritization. This is likely because habitats for these species are extensive, making it difficult to reach full protection without covering a larger area (see https://eclive.shinyapps.io/sweconnect/). These species also have different habitat preferences from the other species, particularly the goby species, which can occur in deeper and more exposed areas. As such, in the initial expansion of the MPA network, reaching targets for these species would incur a large shortfall for many of the other species, which are more spatially congruent Figure 5.

## DISCUSSION

### Conservation planning for habitat representation, connectivity, and vulnerability

One of the greatest challenges in SCP is the need to consider a broad range of ecological and socioeconomic factors that might influence biodiversity outcomes in response to protection. Nonetheless, MPA networks must be designed to ensure not only that the full range of biodiversity is represented but also that the habitats in the network are well connected and that vulnerable conservation features are prioritized to mitigate potential losses of biodiversity. Our approach to SCP incorporates all of these concepts for the assessment of MPA networks and for determining priority areas for future protection. A key contribution of this approach to SCP is the consideration of pressures from human activities on connectivity processes. Within conservation planning, a disproportionate focus has been given to reaching targets for ecological features (e.g., representation of habitats) or processes (e.g., connectivity), and relatively little attention has been given to specifically incorporating targets related to human activities and the pressures they impose on ecosystems (Pressey et al., 2021). Failure to consider pressures on ecosystems in conservation planning can lead to MPA networks that do not have a positive conservation impact because MPAs are placed in locations where habitats and ecosystems are unlikely to be disturbed in the future (Devillers et al., 2015, 2020).

Spatially quantifying ecological connectivity can be challenging and complex, so it is often neglected in spatial conservation planning (Beger et al., 2022). These challenges are compounded when attempting to measure and understand the effects of anthropogenic pressures on connectivity, which requires careful consideration of the unique life‐history stages and attributes of species (Crook et al., 2015). For example, species that disperse primarily through passive larval dispersal may be affected mostly by pressures related to climate change, such as changes in ocean currents, temperature, and salinity (Bashevkin et al., 2020). Species that disperse primarily through active movements in the juvenile or adult phases are likely to be affected more so by direct physical disturbance, such as fishing, dredging, or coastal development. Due to these challenges, there exists a substantial knowledge gap in conservation planning methods for preventing the loss of ecological connectivity in response to anthropogenic pressures (but see Kang et al., 2024).

The incorporation of connectivity in spatial prioritization typically involves a trade‐off between simplistic approaches (e.g., prioritizing spatially clustered habitats) and complex, computationally demanding approaches (e.g., incorporation of connectivity matrices). Complex approaches, such as those provided by the Marxan Connect software (Daigle et al., 2020), are useful but are generally limited to small areas or low resolution. Our approach to spatial prioritization, in which habitat connectivity and vulnerability to isolation are quantified spatially, can be incorporated into a variety of conservation decision support tools, such as Marxan (Watts et al., 2009), Zonation (Moilanen et al., 2022), and prioritizr (Hanson et al., 2024), over large extents and at high resolution.

### Adequacy of the Swedish MPA network for conservation of coastal ecosystems

The protection of features in the MPA network was generally better than would be expected from random allocation of MPAs, suggesting that they have been reasonably well placed for habitat representation, maintenance of connectivity, and protection of habitats vulnerable to isolation. However, there is a concerning lack of protection of these features in strict MPAs. Not only is strict protection of these features poor, but there was also evidence of a negative bias toward avoiding strict protection of these features. An assessment performed in 2011 on the representation of fish habitats in the Stockholm and Åland archipelagos by Sundblad et al. (2011) showed that the MPA network at that time performed slightly worse than random. Our results provide some indication that coastal fish species have become somewhat better represented in the last decade, though protection levels for many biodiversity features included in this analysis are still well below the widely accepted ecologically necessary level of 30%. Importantly, protection levels for some of the ecologically important top predator species, such as perch and pike, were among the poorest, particularly in terms of vulnerability to isolation and connectivity decline. However, our analyses included only a subset of Baltic Sea fish species, and some ecologically and economically important species could not be included, such as cod, herring, flounder, and salmon, and no invertebrates or plants were included at all.

Poor representation of features, particularly in strict MPAs, is likely a result of insufficient knowledge and spatial data at the time of planning, insufficient consideration of national biodiversity patterns when planning at subnational levels, and competing interests between conservation and economically important activities. Of particular importance is a likely bias in protection away from marine areas used for human activities. In the Baltic Sea, and in many other regions of the world, areas attractive for human use tend to be high in biodiversity (Araújo, 2003), particularly when human activities derive value from resources related to biodiversity (e.g., fishing, recreation, and tourism [Hansen et al., 2019; Sundblad & Bergström, 2014]). This creates a dilemma in which impactful conservation necessitates economic sacrifices from people and industries extracting value from marine ecosystems. However, the implementation of MPAs can have negative socioeconomic consequences, such as the opportunity costs of protection caused by displacing marine activities that humans rely on for livelihoods and well‐being. In practice, consideration of these factors could reduce negative social consequences and will likely lead to greater compliance and community support. Nonetheless, protection of crucial habitats supporting connectivity processes is likely to produce disproportionate benefits to biodiversity across the region by preventing the loss of habitats exporting a large number of juveniles and adults to unprotected habitats, thereby enhancing their resilience to disturbance events from human activities and broader pressures, such as climate change and eutrophication.

### Priorities for strengthening and expanding MPA networks

Nations around the world are now in the process of implementing policies and plans for the expansion of MPA networks as part of the global 30×30 initiative in the Kunming–Montreal Global Biodiversity Framework. In the European Union, member states have now committed to the protection of 30% of sea (and land) areas by the year 2030 as part of the EU Biodiversity Strategy for 2030 (European Commission, 2021). In the coming years, it is crucial not only that MPA networks are expanded but also that new MPAs are placed in locations that provide the greatest possible benefits to biodiversity. Decisions by parties to the Convention on Biological Diversity and the Kunming–Montreal Global Biodiversity Framework have also set explicit targets for the prioritization of areas that contribute to connectivity and protection of vulnerable habitats and species (CBD, 2008; Watson et al., 2023). In national and regional policies, these aspects of biodiversity and ecosystem importance are often considered key criteria for determining the ecological coherence of PA networks (Berkström et al., 2022).

Using coastal fish species in the Swedish Baltic Sea as an example, we demonstrated an approach to identifying priority areas for the expansion of MPA networks to efficiently conserve species habitats and connectivity. We showed how the degree to which connectivity is threatened by anthropogenic disturbance can be spatially quantified and how these data can be used in conservation decision support tools. Furthermore, we show how these data can be used to both assess existing PA networks and identify candidate sites for new PAs. The priority areas we identified provide excellent candidates for new MPAs in Sweden that ensure the network is representative of important species, is well connected, and will effectively mitigate pressures on biodiversity. Full protection of all of the species and features included in our analyses could be achieved by expanding the MPA network to cover 20% of the study region, and a mean protection of 90% could be achieved by expanding it to 15% of the study region. However, it may be more challenging to reach such efficient solutions when species, habitats, and connectivity processes are more widespread or spatially incongruous. Nonetheless, we found that even small expansions of the MPA network drastically improved the protection of biodiversity, which has also been shown in other study regions (Virtanen et al., 2018).

The 30×30 agreement also includes objectives for 10% of the sea to be strictly protected. In our study area, only 2.8% were in strict MPAs, which means that to meet these objectives Sweden will need to designate many new strict MPAs or will need to upgrade existing MPAs. However, many of the MPAs we defined as strict do not explicitly prohibit fishing activities. As such, this is likely an overestimate of the amount of strict protection in the region, particularly in relation to the conservation of fish species, depending on how strict protection is defined. The high‐priority areas we identified are excellent candidates for the establishment of strict MPAs over the coming years. Aside from the specific spatial locations identified in our analysis, as a general guideline, we suggest implementing new strict MPAs in shallow coastal areas. Shallow coastal areas are not only important for the connectivity of fish species but also exposed to substantial pressures from human activities, such as dredging, boating, fishing, and coastal development (Hansen et al., 2019; Sundblad & Bergström, 2014). In addition, conservation efforts should focus on important coastal predator species, such as perch and pike, which have relatively poor coverage in the existing MPA network and play a crucial role in maintaining ecosystem function through moderation of trophic cascade effects (Eklöf et al., 2020; Olin et al., 2024).

### Ways forward

Our method is applicable to a variety of contexts but could be adapted or improved according to national policies or the social and ecological context of different areas. For example, in marine regions where connectivity is driven mainly by larval dispersal, biophysical models based on hydrodynamics are necessary (e.g., Jonsson et al., 2016). These methods could also be improved in future analyses by the collection of empirical data on how anthropogenic pressures affect connectivity, for which information is extremely limited (Berkström et al., 2022). Due to the lack of empirical data on which to base the models, we have relied on various assumptions related to these interactions. However, with sufficient data, further analyses could develop explicit models of population dynamics to determine absolute outcomes for species populations (e.g., in terms of biomass or abundance). Furthermore, species‐specific measures of population dynamics can be applied with some methods, such as eigenvalue perturbation theory, to identify important areas for connectivity (Jacobi & Jonsson, 2011), but, due to computational requirements, these are currently limited to simpler connectivity networks at lower resolutions. Nonetheless, as nations across the world push to expand PA networks in the hopes of achieving 30×30 targets, it is essential that spatial prioritization for conservation planning consider the broad range of ecological and socioeconomic factors that influence the persistence of natural ecosystems.

## Supporting information



Supporting information
