# Supplementary material for: Identifying priority areas for conservation to promote connectivity and mitigate the impacts of anthropogenic disturbance
Source: Conserv Biol. 2025 May 31;39(6):e70083. doi: 10.1111/cobi.70083 (PMC12658951; doi:10.1111/cobi.70083)
Supplement: Supplementary file 1 — Supporting information [file COBI-39-e70083-s001.docx]

**Appendix S1 – Supporting tables and figures**

**Table S1.1** List of species included in the analysis and spatial prioritization. Included are the assumed dispersal distances and justification for the assumption.

| **Scientific name** | **Common name** | **Dispersal distance (km)** | **Justification** |
| --- | --- | --- | --- |
| *Perca fluviatilis* | Perch | 10 | Assumed 10 km based on Berkström et al. (2022) & Saulamo & Neuman (2002). |
| *Abramis brama* | Common bream | 10 | Assumed 10 km based on Berkström et al. (2019), who stated "several km". A maximum dispersal distance of 10 km supported by Saulamo & Neuman (2002). |
| *Phoxinus phoxinus* | Common minnow | 10 | Assumed 10 km based on similarity to other cyprinids. |
| *Esox lucius* | Pike | 5 | Assumed 5 km based on Berkström et al. (2022) & Saulamo & Neuman (2002). |
| *Gymnocephalus cernuus* | Ruffe | 15 | Assumed 15 km based on Berkström et al. (2022). |
| *Sander lucioperca* | Zander | 10 | Assumed 10 km based on Berkström et al. (2022) & Saulamo & Neuman (2002). |
| *Leuciscus idus* | Ide | 10 | Assumed 10 km based on similarity to other cyprinids. |
| *Alburnus alburnus* | Common bleak | 10 | Assumed 10 km based on similarity to other cyprinids. |
| *Rutilus rutilus* | Roach | 10 | Assumed 10 km based on similarity to other cyprinids. |
| *Carassius carassius* | Crucian carp | 10 | Assumed 10 km based on similarity to other cyprinids. |
| *Scardinius erythrophthalmus* | Common rudd | 10 | Assumed 10 km based on similarity to other cyprinids. |
| *Pomatoschistus flavescens* | Two-spotted goby | 10 | Assumed 10 km based on the species typical habitat and behaviour. All relatively sedentary species inhabiting shallow coastal areas were assumed to have a maximum dispersal distance of 10 km. |
| *Pungitius pungitius* | Nine-spined stickleback | 10 | Assumed 10 km based on the species typical habitat and behaviour. All relatively sedentary species inhabiting shallow coastal areas were assumed to have a maximum dispersal distance of 10 km. |
| *Pomatoschistus minutus* | Sand goby | 10 | Assumed 10 km based on the species typical habitat and behaviour. All relatively sedentary species inhabiting shallow coastal areas were assumed to have a maximum dispersal distance of 10 km. |
| *Tinca tinca* | Tench | 10 | Assumed 10 km based on similarity to other cyprinids. |
| *Gobius niger* | Black goby | 10 | Assumed 10 km based on the species typical habitat and behaviour. All relatively sedentary species inhabiting shallow coastal areas were assumed to have a maximum dispersal distance of 10 km. |

| **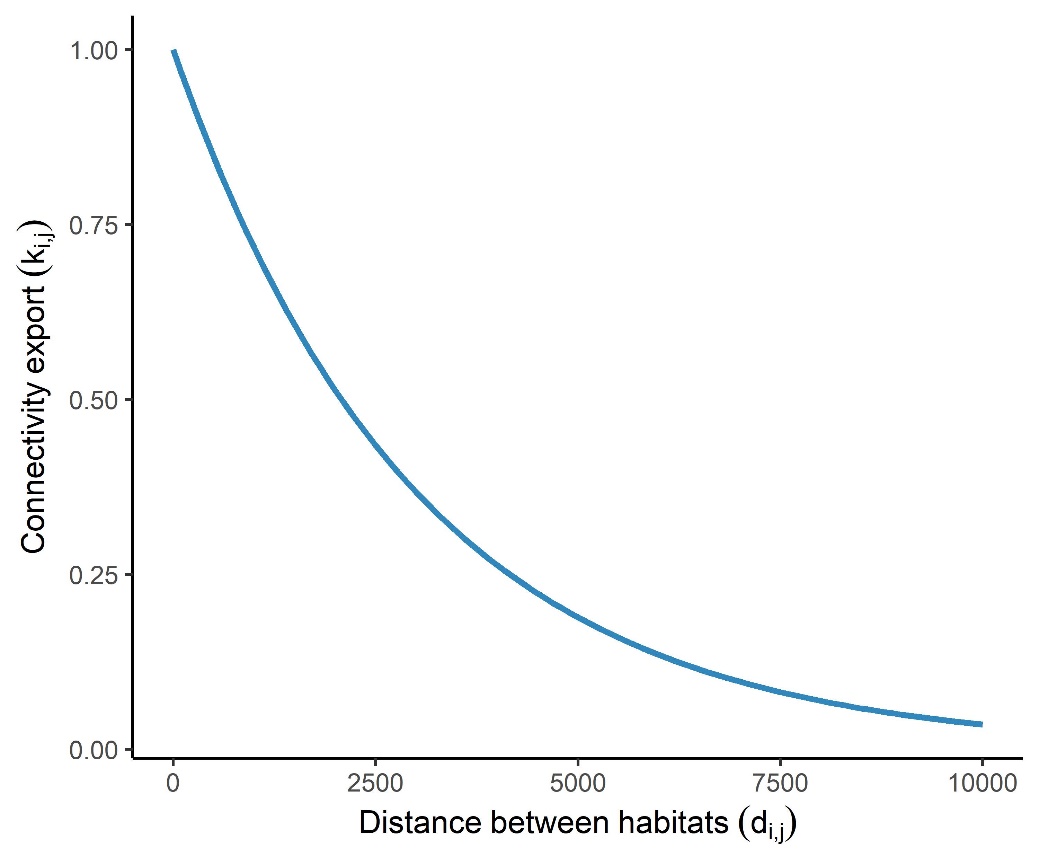** |
| --- |
| **Figure S1.2** The dispersal kernel describing the relationship between *d_i,j_*, the distance between habitat cells along the least cost path, and *k_i,j_*, the connectivity export from habitat cell *i* to habitat cell *j*. A full description of the connectivity model is provided in Equation 1. |

| **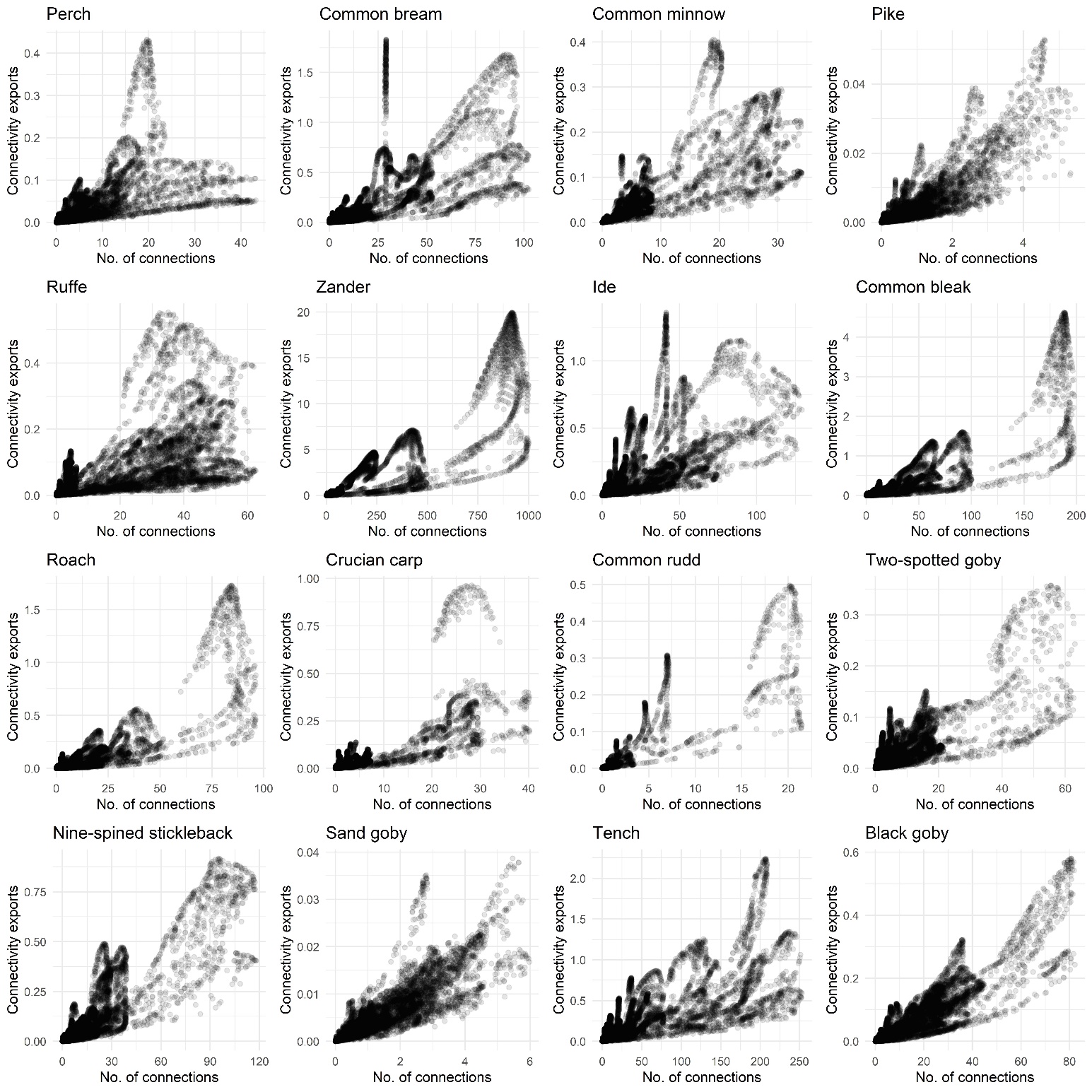** | | |
| --- | --- | --- |
| **Figure S1.3** The relationship between the number of connections (×10^-7^) between each habitat cell to other habitats and total connectivity exports (×10^-7^) for each species over three generations. For plotting purposes, a random subset of ten thousand habitats were selected for species containing over ten thousand habitats cells. |  |  |

**References**

Berkström C, Wennerström L, Bergström U. 2022. Ecological connectivity of the marine protected area network in the Baltic Sea, Kattegat and Skagerrak: Current knowledge and management needs. Ambio 51:1485–1503.

Saulamo K, Neuman E. 2002. Local management of Baltic fish stocks – significance of migrations. Pages 1–18. 2002:9, Fiskeriverket Informerar.
